# Synthesis and characterization of novel bioactive 1,2,4-oxadiazole natural product analogs bearing the *N*-phenylmaleimide and *N*-phenylsuccinimide moieties

**DOI:** 10.3762/bjoc.9.259

**Published:** 2013-10-25

**Authors:** Catalin V Maftei, Elena Fodor, Peter G Jones, M Heiko Franz, Gerhard Kelter, Heiner Fiebig, Ion Neda

**Affiliations:** 1 Institut für Anorganische und Analytische Chemie, Technische Universität Carola Wilhelmina, Hagenring 30, D-38106 Braunschweig, Germany; 2 InnoChemTech GmbH, Hagenring 30, D-38106 Braunschweig, Germany; 3 Oncotest GmbH, Am Flughafen 12–14, 79108 Freiburg, Germany; 4 Institutul National de Cercetare Dezvoltare pentru Electrochimie si Materie Condensata, Str. Dr. A. Paunescu Podeanu Nr. 144, Ro-300569 Timisoara, Romania

**Keywords:** antitumor activity, bioisosteres, maleimide, natural product analogs, 1,2,4-oxadiazoles

## Abstract

Taking into consideration the biological activity of the only natural products containing a 1,2,4-oxadiazole ring in their structure (quisqualic acid and phidianidines A and B), the natural product analogs 1-(4-(3-*tert*-butyl-1,2,4-oxadiazol-5-yl)phenyl)pyrrolidine-2,5-dione (**4**) and 1-(4-(3-*tert*-butyl-1,2,4-oxadiazol-5-yl)phenyl)-1*H*-pyrrole-2,5-dione (**7**) were synthesized starting from 4-(3-*tert*-butyl-1,2,4-oxadiazol-5-yl)aniline (**1**) in two steps by isolating the intermediates 4-(4-(3-*tert*-butyl-1,2,4-oxadiazol-5-yl)phenylamino)-4-oxobutanoic acid (**3**) and (*Z*)-4-(4-(3-*tert*-butyl-1,2,4-oxadiazol-5-yl)phenylamino)-4-oxobut-2-enoic acid (**6**). The two natural product analogs **4** and **7** were then tested for antitumor activity toward a panel of 11 cell lines in vitro by using a monolayer cell-survival and proliferation assay. Compound **7** was the most potent and exhibited a mean IC_50_ value of approximately 9.4 µM. Aniline **1** was synthesized by two routes in one-pot reactions starting from *tert*-butylamidoxime and 4-aminobenzoic acid or 4-nitrobenzonitrile. The structures of compounds **1**, **2**, **4**, **5** and **6** were confirmed by X-ray crystallography.

## Introduction

The five-membered heterocyclic 1,2,4-oxadiazole motif is of synthetic and pharmacological interest. It also forms an important constituent of biologically active compounds including natural products [1]. Sawyer et al. have described such compounds as bioisosteres for amides and esters [2], with the 1,2,4-oxadiazoles showing higher hydrolytic and metabolic stability.

**Figure 1:**
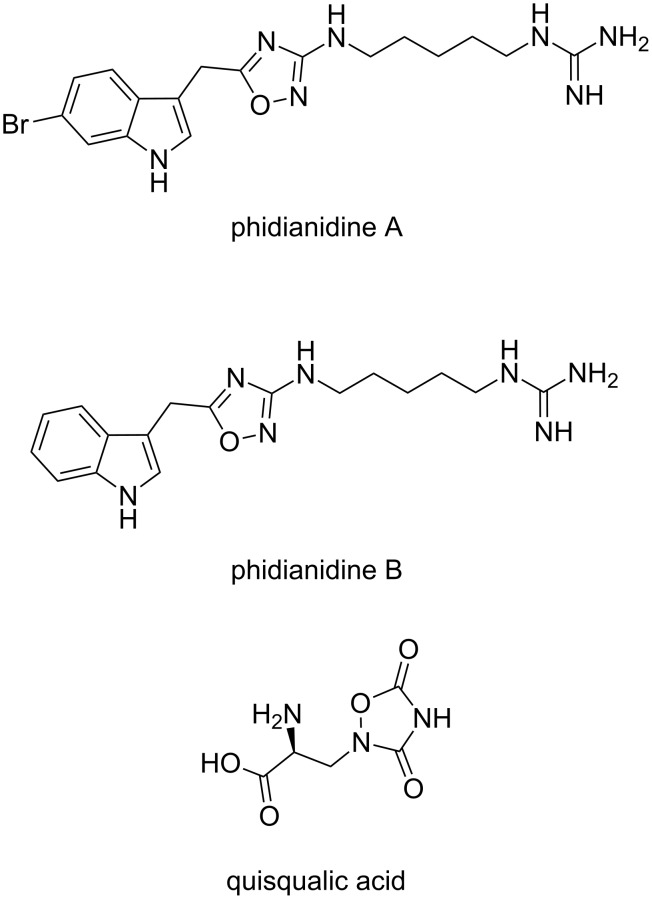
Natural products having a 1,2,4-oxadiazole core.

To the best of our knowledge, there are only a few examples of natural products with a 1,2,4-oxadiazole core or a structure based on it. The 3-substituted indole alkaloids, phidianidines A and B (Figure 1), have been isolated by Carbone et al. from the aeolid opisthobranch *Phidiana militaris* [3]. They are selective inhibitors of the dopamine transporter DAT and partial agonists of the μ opioid receptor [4]. Moreover, these selective molecules are attractive as CNS targets because neither phidianidine A nor B is cytotoxic. Another example of a natural product with a oxadiazole core is quisqualic acid (Figure 1). This metabolite was obtained from the seeds of *Quisqualis indica* and *Q. fructus* [5–6] and is a strong agonist for AMPA (α-amino-3-hydroxy-5-methyl-4-isoxazolepropionic acid) receptors and group I metabotropic glutamate receptors [7].

**Figure 2:**
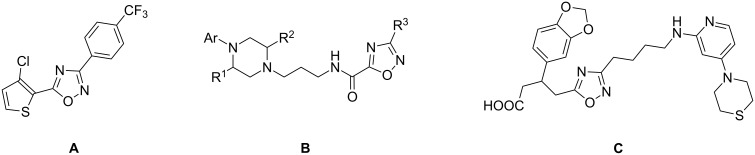
Examples of 1,2,4-oxadiazole antitumorals.

Furthermore, 1,2,4-oxadiazoles are widely used in synthetic chemistry, e.g., in the search for antitumor agents. Cancer consists of more than one hundred different diseases, all of which are characterized by the uncontrolled growth and spread of abnormal cells. In this context, the identification of drugs acting as apoptosis inducers represents an attractive approach for the discovery of new anticancer agents. 1,2,4-oxadiazole **A** (Figure 2) was found to act as an apoptosis agent by a high-throughput screening (HTS) assay [8]. A series of 1,2,4-oxadiazole-5-carboxamides **B** have been synthesized and tested as inhibitors of the glycogen synthase kinase 3 (GSK-3), a key regulator of both differentiation and cellular proliferation [9].

An alternative antitumor strategy involves the inhibition of processes involved in tumor growth, e.g., angiogenesis. Integrin α_v_β_3_ is a receptor that has been found on the surface of many tumor cells and recognizes the arginine–glycine–aspartic acid (RGD) sequence. Antagonists of this receptor are able to inhibit angiogenesis. 1,2,4-Oxadiazolebutanoic acids such as **C** were tested as non-peptidic analogs of α_v_β_3_ antagonists [10]. Furthermore, substituted 1,2,4-oxadiazoles have been described as antirhinovirals [11], benzodiazepine receptor partial agonists [12], anti-inflammatory [13], muscarinic agonists [14], serotoninergic (5-HT_3_) antagonists [15], and growth hormone secretagogues [16].

The maleimide motif is also a useful five-membered heterocycle in pharmacological chemistry. Kratz et al. synthesized maleimide derivatives of doxorubicin and camptothecin. After intravenous administration these designed anticancer drugs bind rapidly to circulating albumin [17–19]. Endogenous albumin could be seen as a drug carrier, as it accumulates in solid tumors according to the pathophysiology of tumor tissue [20–21]. Therefore, designed prodrugs have a higher antitumor efficacy in vivo than drugs. Furthermore, maleimides possess strong antifungal activities against important human opportunistic pathogenic fungi. These antifungal drugs appear to be excellent candidates for further development [22–27]. Barrett et al. point out that the possibility of performing chemical modifications is a requirement for developing novel drugs, a strong activity is just the starting point [28].

Another moiety worth investigation is succinimide, because *N*-phenylsuccinimides are regarded as some of the most efficacious agricultural fungicides [29–30]. They have also been shown to be selective nephrotoxic compounds [31–32].

Considering natural products with the 1,2,4-oxadiazol moiety, such as phidianidines A and B (selective inhibitors of DAT), we decided to synthesize, isolate and characterize novel natural product analogs of 1,2,4-oxadiazole derivatives bearing *N*-phenylmaleimide or *N*-phenylsuccinimide functionalities in order to improve their biological activity. The new derivatives have been tested for in vitro antitumor activity toward a panel of 11 cell lines.

## Results and Discussion

Clapp reviewed the synthesis of 1,2,4-oxadiazoles [33]. He pointed out that two general methods dominate the practical preparation (≈95%):

(a) The condensation of amidoximes with carboxylic acid derivatives.

(b) The dipolar cycloaddition of nitrile oxides to nitriles.

**Scheme 1:**
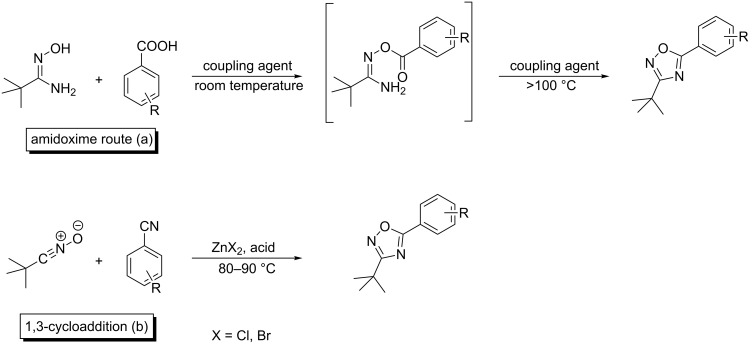
Common synthetic strategies toward 1,2,4-oxadiazoles; (a) amidoxime route; (b) 1,3 dipolar cycloaddition route.

The general approach for the synthesis of 1,2,4-oxadiazoles is illustrated in Scheme 1.

Using route (a), the amidoxime route, the carboxylic acid has to be employed in an activated form. The activated carboxylic acid can be prepared beforehand or in situ by several methods [34], e.g., as an acyl chloride or by the use of *N*,*N*′-carbonyldiimidazole (CDI). In the first step the amidoxime is *O*-acylated with the activated derivative in a condensation reaction. The *O*-acylated amidoxime can be isolated or it can immediately undergo the cyclisation to the heterocyclic oxadiazole ring. This cyclodehydration reaction takes place by heating to temperatures above 100 °C [35–36]. Microwave techniques have also been employed in the synthesis of such heterocycles. The advantage of CDI is that it activates the carboxylic acid in situ and can be used for step 1 and step 2 in DMF.

**Scheme 2:**
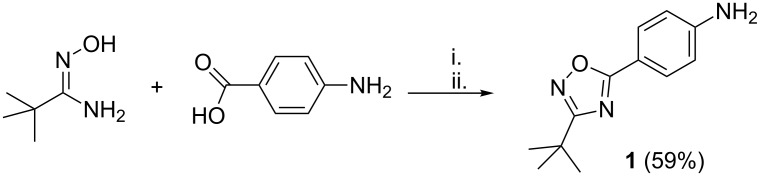
One-pot synthesis of 4-(3-*tert*-butyl-1,2,4-oxadiazol-5-yl)aniline (**1**) by using the amidoxime route. i. 1.1 equiv CDI in DMF, 30 minutes; ii. 1.1 equiv CDI in DMF, 120 °C, 4 h.

Scheme 2 presents the one-pot synthesis of 4-(3-*tert*-butyl-1,2,4-oxadiazol-5-yl)aniline (**1**) starting from *tert*-butylamidoxime and 4-aminobenzoic acid. Activation of the 4-aminobenzoic acid with CDI and further acylation of the *tert*-butylamidoxime with DMF as a solvent furnished the *O*-acylamidoxime, which was not isolated. After heating to 120 °C for four hours, it underwent a cyclodehydration reaction, delivering aniline **1** with 59% yield after purification.

**Figure 3:**
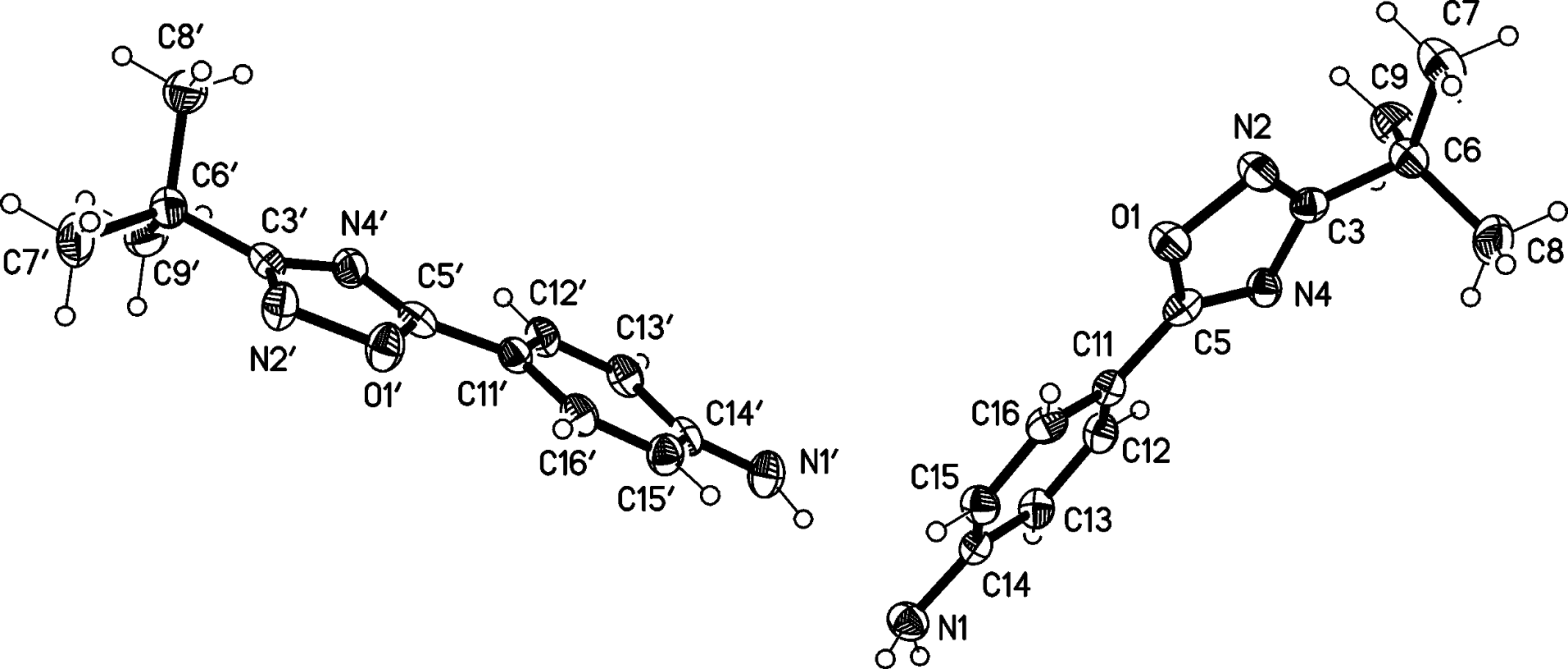
Molecular structure of 4-(3-*tert*-butyl-1,2,4-oxadiazol-5-yl)aniline (**1**). Atoms are drawn as 50% thermal ellipsoids. One hydrogen at N1’ is eclipsed.

**Figure 4:**
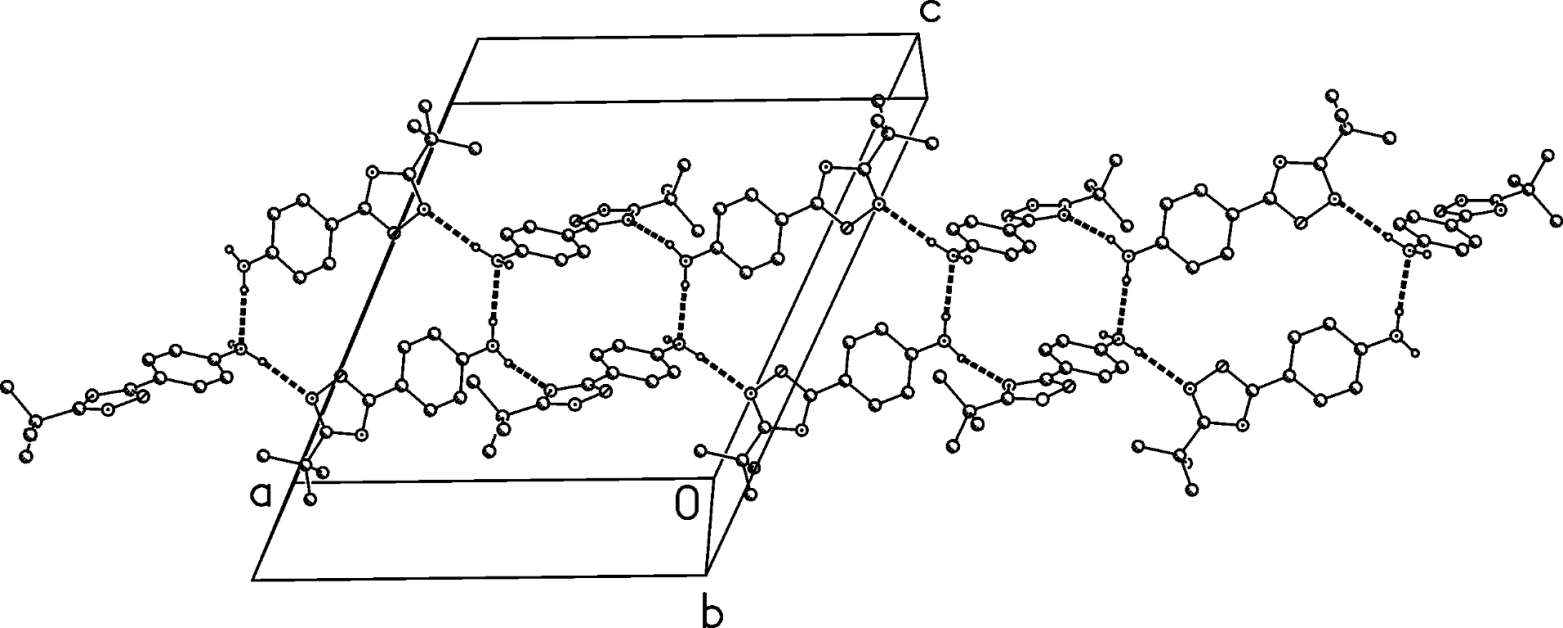
Packing diagram of compound **1**. Hydrogen bonds are indicated as dashed lines.

The structure of compound **1** was confirmed by X-ray structure determination (Figure 3 and Figure 4). It crystallizes with two molecules in the asymmetric unit, which differ in the relative orientation of the rings (interplanar angles 22° and 9°). Three of the four NH hydrogens are involved in hydrogen bonds, leading to ribbons of H-bonded rings parallel to the *a* axis.

Following the second route, the 1,3-dipolar cycloaddition, with the purpose of increasing the yield of compound **1**, we used *p*-toluenesulfonic acid (PTSA)–ZnCl_2_ as a catalyst for the synthesis of aniline **1** from amidoximes and organic nitriles [37]. *tert*-Butylamidoxime and 4-aminobenzonitrile were mixed in DMF with catalytic amounts of PTSA–ZnCl_2_. First, *tert*-butylamidoxime is activated by PTSA–ZnCl_2_; resulting in the formation of a Lewis acid–ammonia complex as a leaving group, giving rise to the formation of the nitrile oxide. The 1,2,4-oxadiazole moiety is established by the 1,3-dipolar cycloaddition of nitrile oxide to the 4-aminobenzonitrile. However, the Lewis acid might also be involved in the formation of the heterocycle via a Lewis acid catalyzed [3 + 2] cycloaddition reaction. Unfortunately, the yield for this reaction was very low (<20%).

**Scheme 3:**
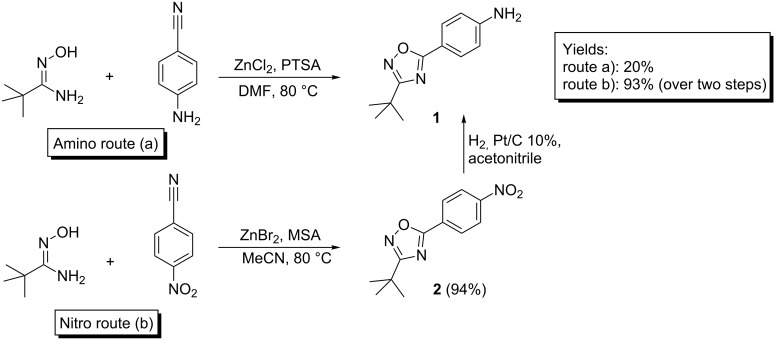
One-pot synthesis of 4-(3-*tert*-butyl-1,2,4-oxadiazol-5-yl)aniline (**1**) by using the 1,3-dipolar cycloaddition route.

In order to obtain compound **1** by the 1,3 dipolar cycloaddition route we changed the protocol. We synthesized the nitro derivative **2** (3-*tert*-butyl-5-(4-nitrophenyl)-1,2,4-oxadiazole) in situ by using the same catalyst pair as in the first synthesis route. Then, we attempted to hydrogenate nitro compound **2** to the corresponding amine **1** in the same reaction pot without having to isolate the intermediate. The overall yield in this case was 64%. The intermediate **2** was isolated to be fully charazterized. After a series of tests by using various acids as catalyst (*p*-toluensulfonic acid (PTSA), 2-mesitylenesulfonic acid (MSA) and methanesulfonic acid (MeSA) in combination with ZnCl_2_ and ZnBr_2_), MSA–ZnBr_2_ in acetonitrile proved to be the best combination for the preparation of compound **2** from *tert*-butylamidoxime and 4-nitrobenzonitrile under mild conditions. The results are summarized in Table 1. The optimized yield was 93% (Scheme 3) which makes this route more practical than the amidoxime route presented in Scheme 2 with a yield of only 59%.

**Table 1:** Optimal conditions for the 1,3-dipolar cycloaddition route.^a^

Entry	Catalyst 1	Catalyst 2	Solvent	Time (h)	Yield (%)

1	PTSA	ZnCl_2_	DMF	5	64
2	MSA	ZnCl_2_	DMF	3	76
3	MeSA	ZnCl_2_	DMF	12	56
4	PTSA	ZnBr_2_	DMF	5	70
5	MSA	ZnBr_2_	DMF	3	79
6	MeSA	ZnBr_2_	DMF	12	58
7	PTSA	ZnCl_2_	MeCN	2	82
8	MSA	ZnCl_2_	MeCN	2	92
9	MeSA	ZnCl_2_	MeCN	12	63
10	PTSA	ZnBr_2_	MeCN	2	86
11	MSA	ZnBr_2_	MeCN	2	93
12	MeSA	ZnBr_2_	MeCN	12	65

^a^General conditions: *tert*-butylamidoxime (1 equiv), 4-nitrobenzonitrile (1 equiv), catalyst 1 (0.3 equiv), catalyst 2 (0.3 equiv), 80 °C.

**Figure 5:**
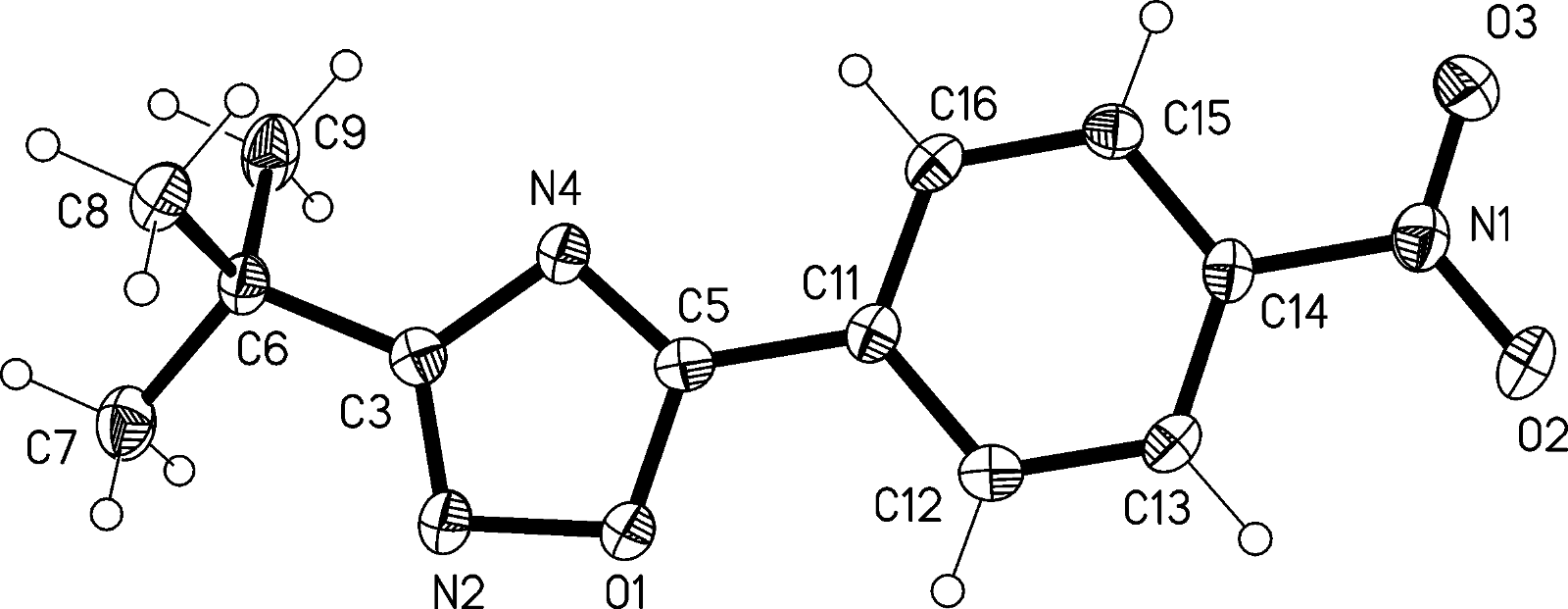
Molecular structure of 3-*tert*-butyl-5-(4-nitrophenyl)-1,2,4-oxadiazole (**2**). Atoms are drawn as 50% thermal ellipsoids.

**Figure 6:**
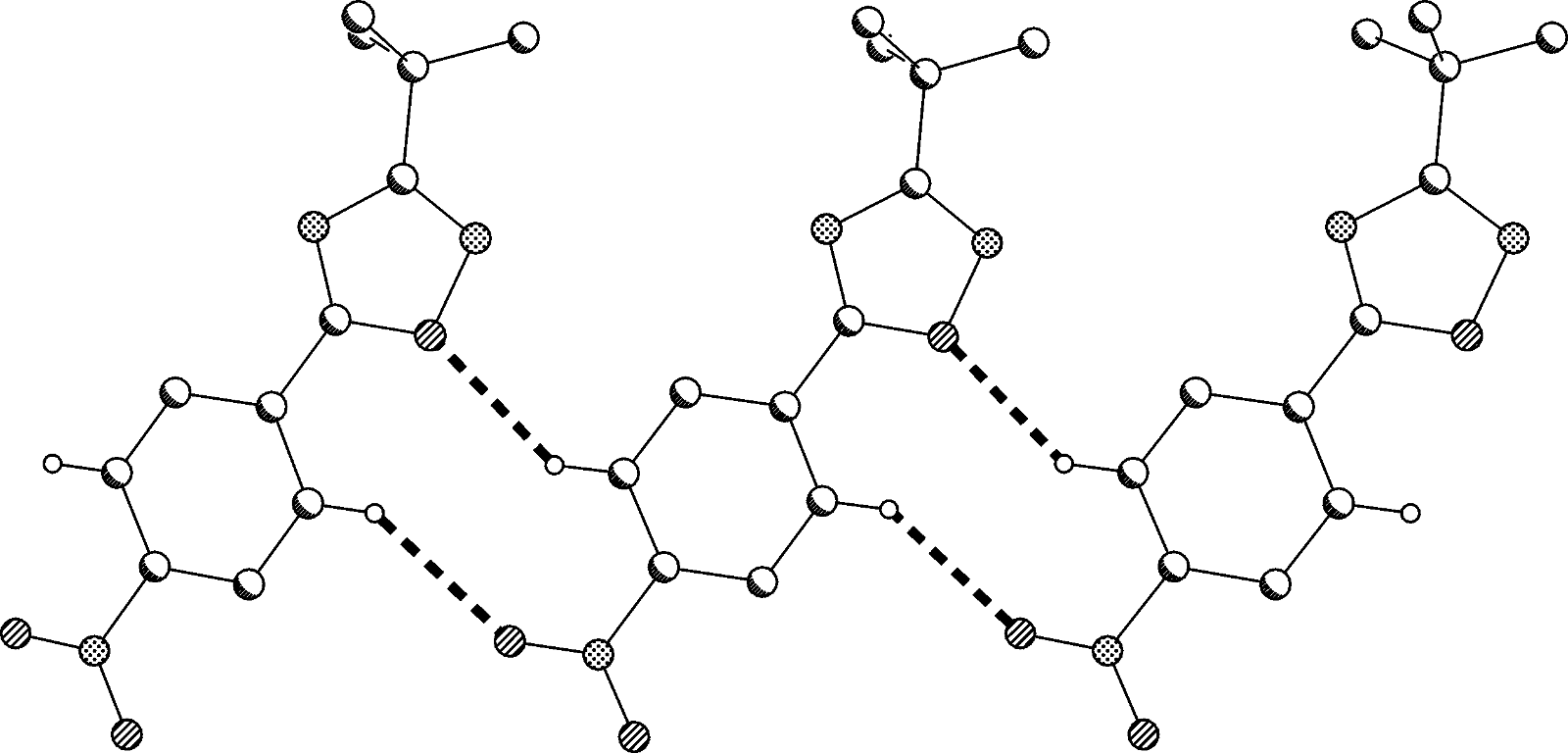
Packing diagram of compound (**2**) showing C–H···O interactions.

The structure of compound **2** was confirmed by X-ray structure determination (Figure 5 and Figure 6). The interplanar angle in compound **2** is only 3° and the molecules are linked to ribbons parallel to the *b* axis by two C–H···O interactions.

Imide derivatives have been found to possess a broad spectrum of biological activities. A variety of methods have been reported for the preparation of this class of compounds. The synthesis follows a two-step protocol. First, it is necessary to synthesize the amide derivative.

**Scheme 4:**
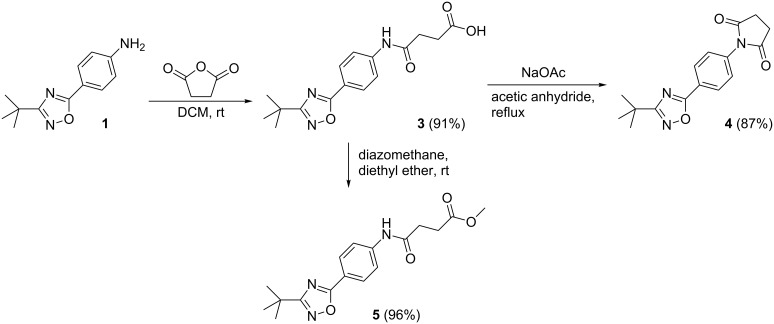
Synthesis of 1-(4-(3-*tert*-butyl-1,2,4-oxadiazol-5-yl)phenyl)pyrrolidine-2,5-dione (**4**).

The synthesis of amide **3** was performed under inert conditions by mixing an equimolar amount of aniline **1** and succinic anhydride in a minimum volume of dichloromethane (Scheme 4). Compound **3** was obtained with a short reaction time and a high yield (91%). The ester derivative methyl 4-(4-(3-*tert*-butyl-1,2,4-oxadiazol-5-yl)phenylamino)-4-oxobutanoate (**5**) was prepared by the addition of a diethyl ether solution of diazomethane to a suspension of amide **3**. The ester **5** was afforded in a quantitative amount.

For the synthesis of imide **4** the starting material, amide **3**, was mixed with an equimolar amount of sodium acetate in acetic anhydride and the mixture was heated for 4 h at 80–85 °C; resulting in the corresponding *N*-arylsuccinimide **4** (87%).

**Figure 7:**
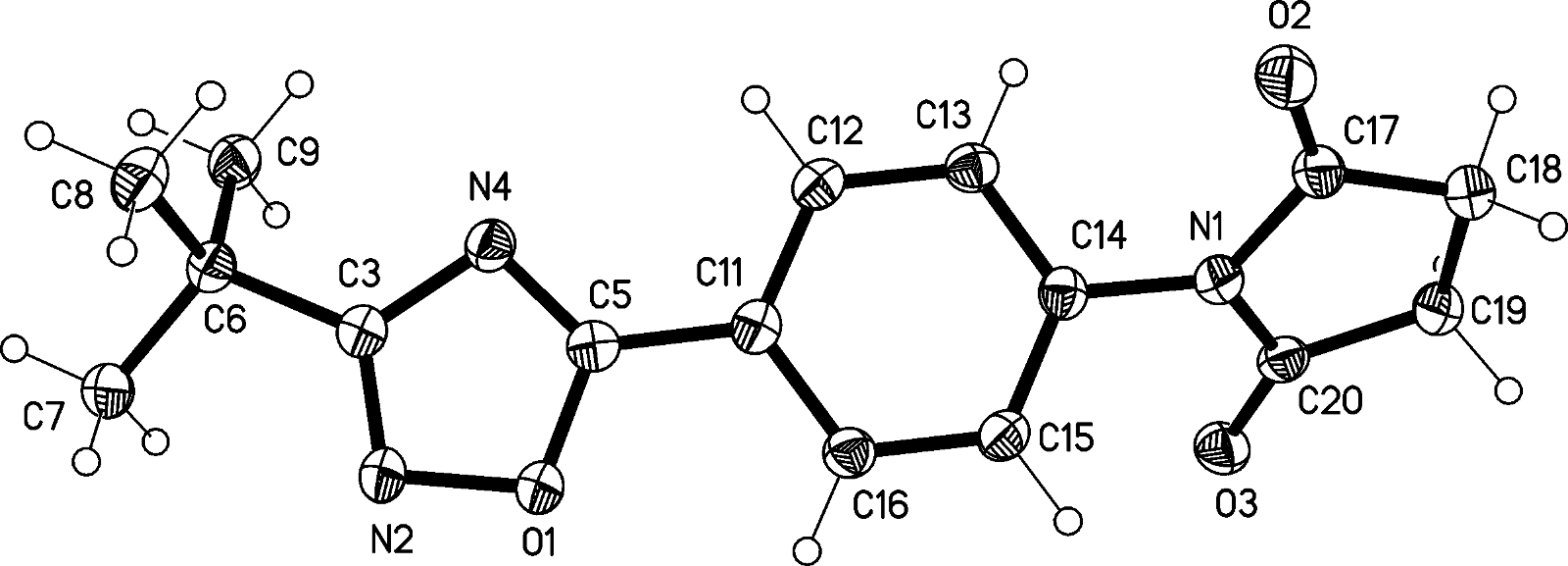
Molecular structure of 1-(4-(3-*tert*-butyl-1,2,4-oxadiazol-5-yl)phenyl)pyrrolidine-2,5-dione (**4**). Atoms are drawn as 50% thermal ellipsoids.

**Figure 8:**
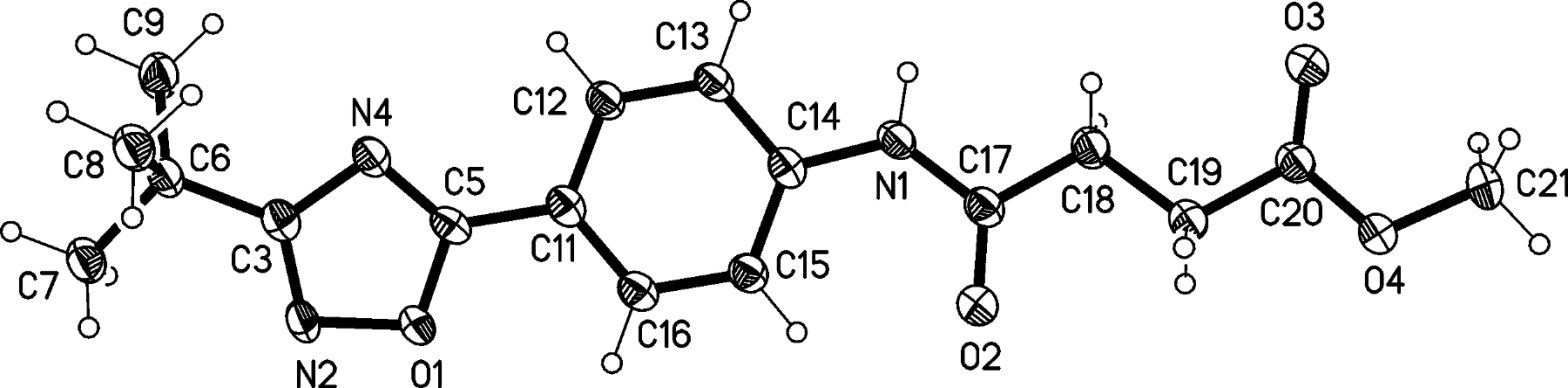
Molecular structure of 4-(4-(3-*tert*-butyl-1,2,4-oxadiazol-5-yl)phenylamino)-4 oxobutanoate (**5**). Atoms are drawn as 50% thermal ellipsoids.

**Figure 9:**
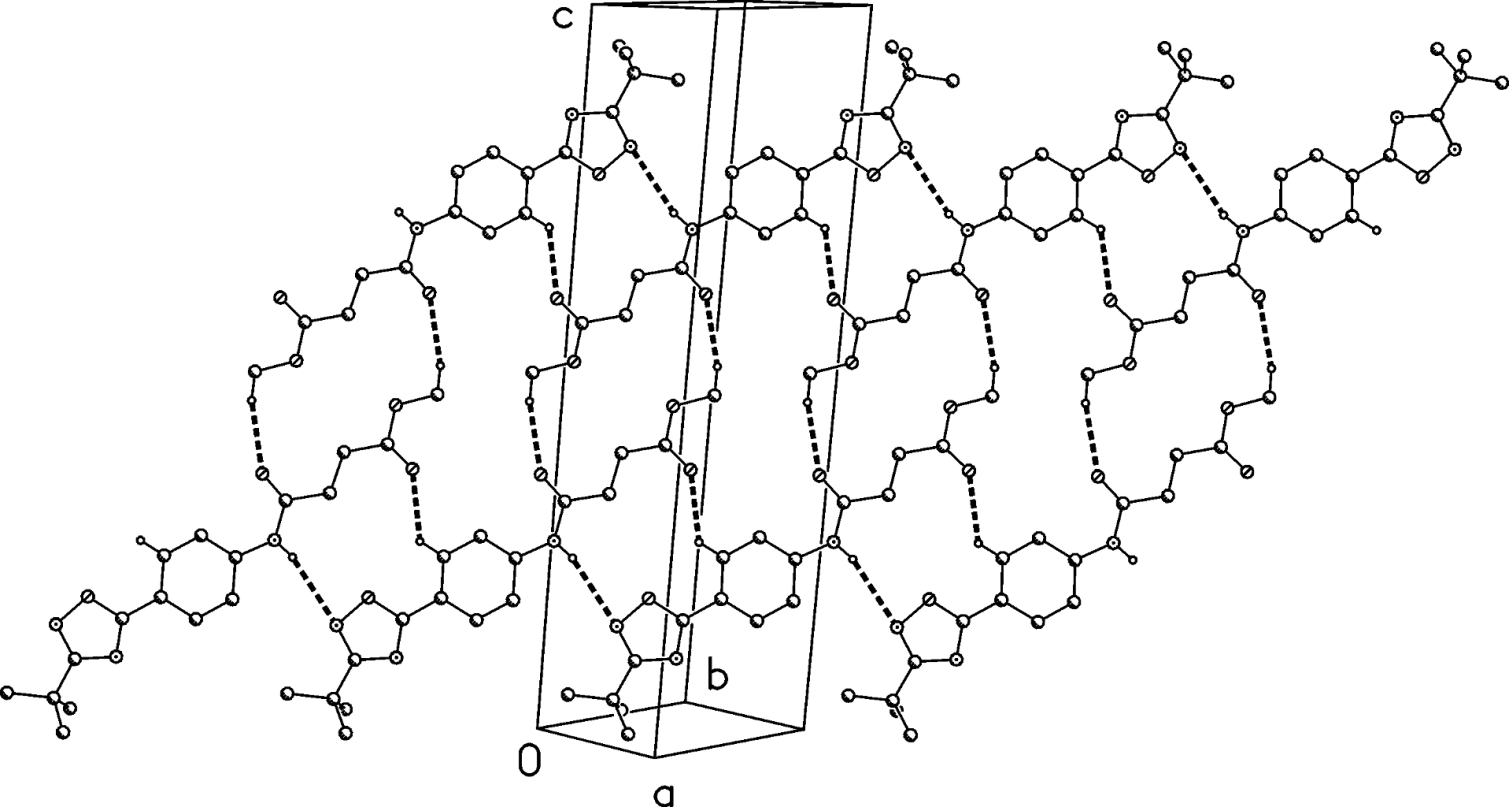
Packing diagram of compound (**5**). Dashed lines indicate hydrogen bonds.

The structures of compounds **4** and **5** were also confirmed by X-ray structure analysis (Figure 7, Figure 8 and Figure 9). In compound **4** the oxazoline and phenyl rings are approximately coplanar (6°), but the pyrrolidine ring is rotated by 52° with respect to the phenyl ring. The main packing interaction is an offset stacking parallel to the *c* axis (not shown). Compound **5** has an interplanar angle of 14°. The molecules are associated into ribbons parallel to [110] by one long N–H···N and two shorter C–H···O interactions.

**Scheme 5:**
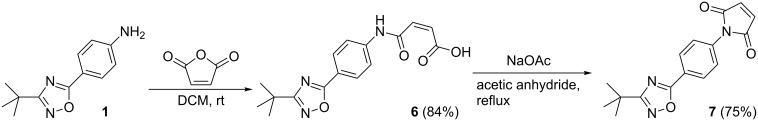
Synthesis of 1-(4-(3-*tert*-butyl-1,2,4-oxadiazol-5-yl)phenyl)-1*H*-pyrrole-2,5-dione (**7**).

The synthesis of amide **6** was performed by mixing an equimolar amount of aniline **1** and maleic anhydride in dichloromethane (Scheme 5). The amide **6** was obtained with a good yield (84%). For the synthesis of imide **7** the amide **6** was mixed with an equimolar amount of sodium acetate in acetic anhydride and the mixture was heated for 4 h at 80–85 °C resulting in the corresponding *N*-arylmaleimide **7** (75%).

**Figure 10:**
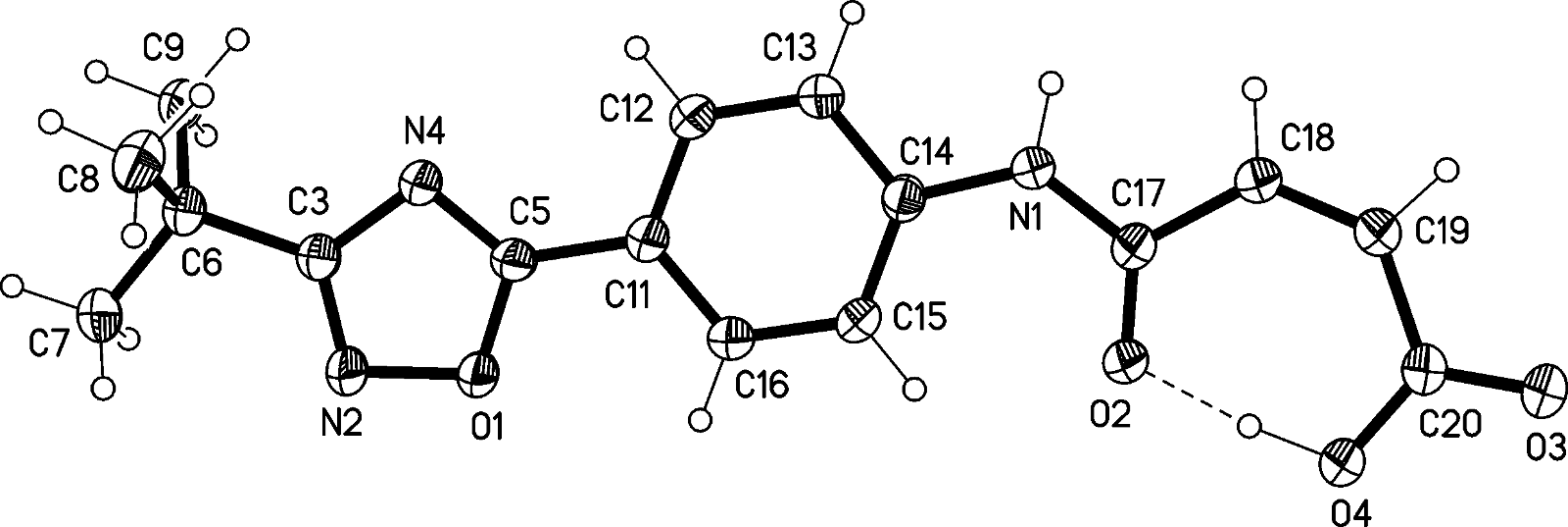
Molecular structure of (*Z*)-4-(4-(3-*tert*-butyl-1,2,4-oxadiazol-5-yl)phenylamino)-4-oxobut-2-enoic acid (**6**). Atoms are drawn as 50% thermal ellipsoids.

**Figure 11:**
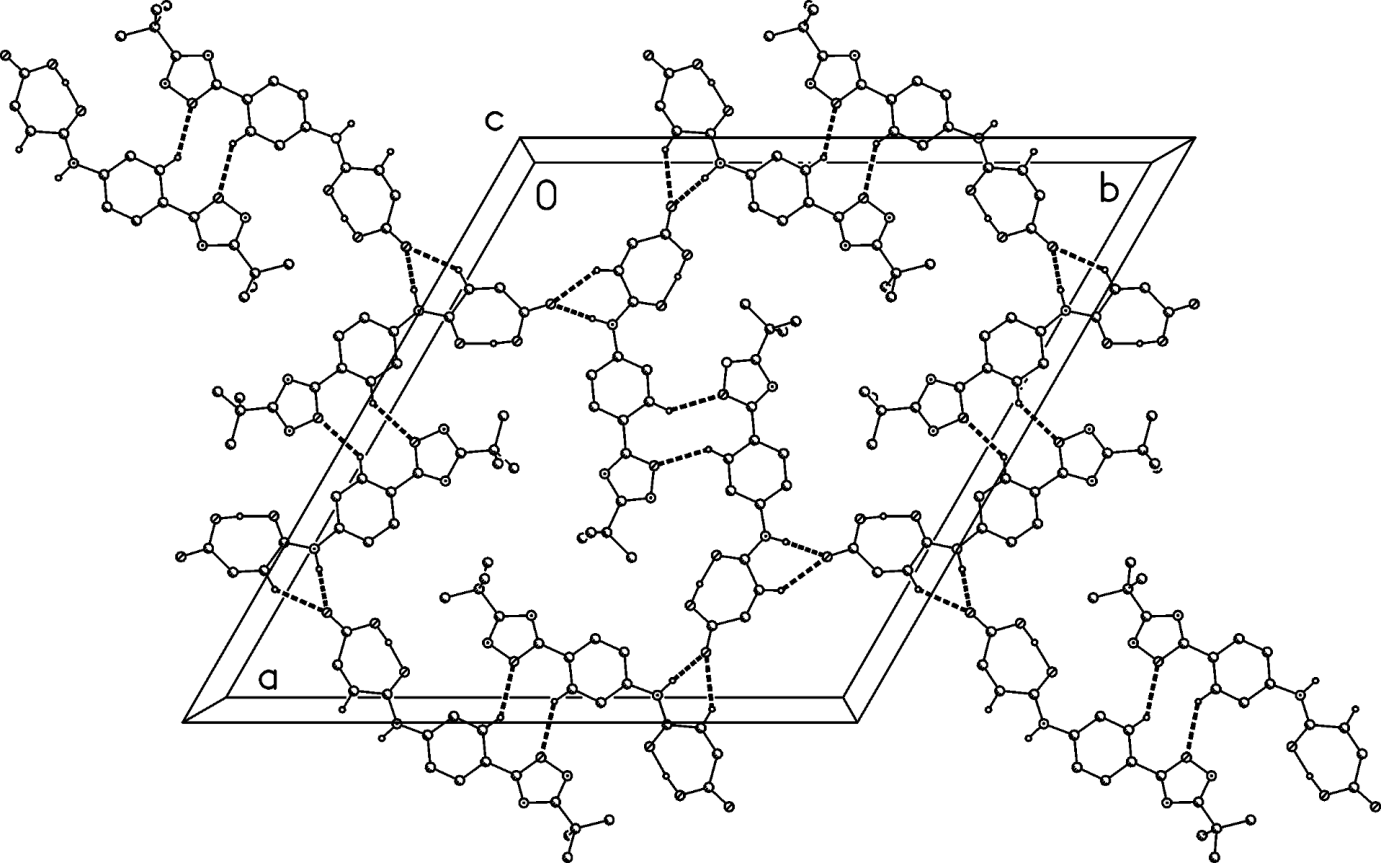
Packing diagram of compound **6**. Dashed lines indicate hydrogen bonds.

The structure of compound **6** was also confirmed by X-ray structure analysis (Figure 10 and Figure 11). In compound **6** the interplanar angle is 11°; the intramolecular hydrogen bond is almost symmetrical (O4–H04 1.03(3), H04···O2 1.47(3)) Å. The molecules are linked to form layers perpendicular to the hexagonal *c* axis by one C–H···O and one three-centre (N–H, C–H)···O interaction. The double bond has a *Z*-configuration.

## Antitumor activity

**Figure 12:**
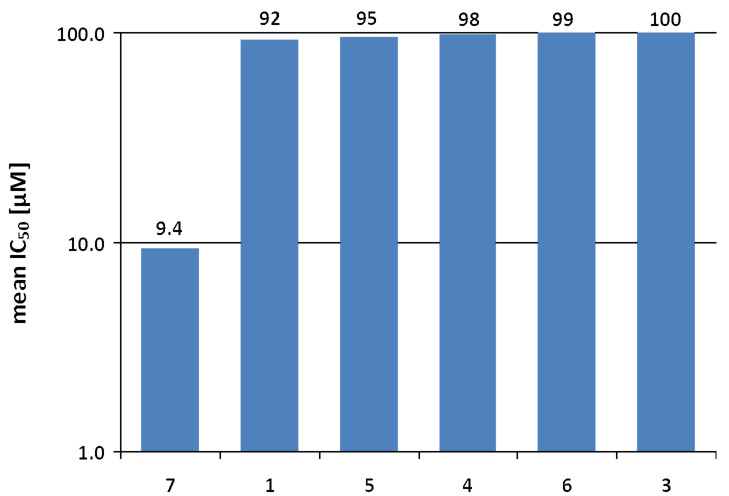
In vitro antitumor activity of compounds **1**, **3**–**7** toward 11 human tumor cell lines.

The in vitro antitumor activity of six synthesized compounds toward a panel of 11 cell lines was assessed by using a monolayer cell survival and proliferation assay. By exhibiting a mean IC_50_ value of 9.4 µM **7** was the most potent compound. Compounds **1**, **5**, **4**, **6** and **3** have only marginal antitumor activity toward the 11 tested cell lines (Figure 12).

**Figure 13:**
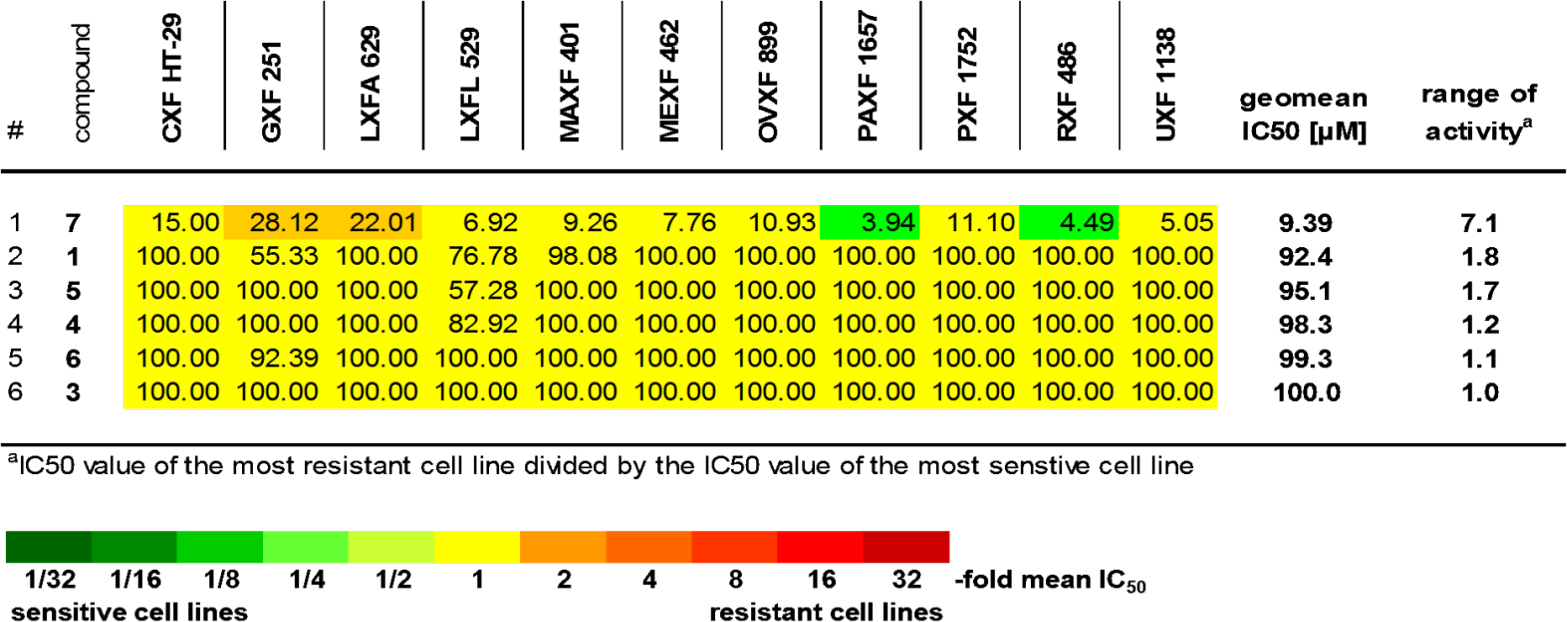
Individual IC_50_ values [µM] of compounds **1**, **3**–**7** in a panel of 11 human tumor cell lines.

An investigation of the activity of compounds in a cell line panel representing various tumor histo-types, as performed in this study, allows the analysis of potency and tumor selectivity and the identification of active compounds which qualify for further preclinical development. Tumor selectivity of the compounds is illustrated in Figure 13 by a heat-map presentation of the individual IC_50_ values. Good tumor selectivity is reflected by cell lines exhibiting above-average sensitivity (i.e., an individual IC_50_ value smaller than 0.5 of the mean IC_50_ value; marked in green) and the range of activity among the cell lines (i.e., the IC_50_ value of the most resistant cell line divided by the IC_50_ value of the most sensitive cell line). Overall, a good antitumor potency (i.e., a mean IC_50_ < 30 µM) combined with a good selectivity (i.e., a range of activity > 8 or at least 2 cell lines exhibiting above-average sensitivity) was obvious for compound **7**.

## Conclusion

Two novel 1,2,4-oxadiazol natural product-inspired derivatives bearing *N*-phenylmaleimide or *N*-phenylsuccinimide moieties were synthesized in two steps starting from 1,2,4-oxadiazole amine **1**. All intermediate derivatives were isolated, characterized and tested in vitro for antitumor activity toward a panel of 11 cell lines by using a monolayer cell survival and proliferation assay. With an IC_50_ value of 9.4 µM, compound **7** was the most active, probably because maleimide is able to rapidly bind to the cysteine-34 position of circulating albumin, transported as an albumin conjugate (macromolecular drug), and then released in the target tissue by acid related cleavage or enzymatic cleavage. Compounds **1**, **3**, **4**, **5** and **6** have no or only marginal activity toward the 11 cell lines tested. Considering the bioactive natural products (quisqualic acid and phidianidines A and B), it is of great pharmacologic interest to synthesize new derivates of 1,2,4-oxadiazole with an efficient biological transport in cells by using natural transporters such as amino acids, peptides or sugars. Moreover, it is desirable to reduce, or even remove completely, the secondary effects to minimize the toxicity and increase the selectivity. All derivatives were obtained with a high purity (at least 97% based on ^1^H NMR) and good to high yields (75–96%). The structural assignments were corroborated by X-ray structure analysis.

## Experimental

### General

All reagents were purchased from commercial sources (Sigma-Aldrich or Acros) and used without further purification. Solvents were of analytical grade. ^1^H and ^13^C NMR spectra were recorded at room temperature on a Bruker Avance 200 operating at 200 MHz for ^1^H and 50 MHz for ^13^C. Chemical shifts (δ) are reported relative to the tetramethylsilane peak set at 0.00 ppm. In the case of multiplets, the signals are reported as intervals. Signals were abbreviated as s, singlet; d, doublet; t, triplet; q, quadruplet; m, multiplet. IR spectra were recorded with a Bruker Vertex 70 ATR. Mass spectra (electronic ionisation, 70 eV) were recorded on a Finnigan MAT 8400-MSS and Finnigan MAT 4515. High resolution mass spectra were recorded on a Finnigan MAT 95 XP. Melting points were measured with a Schropp Gerätetechnik MPM-HV2. The melting points are uncorrected. Column chromatography was carried out by using Merck silica gel 60 (70–200 mesh).

### Procedures

#### Synthesis of 4-(3-*tert*-butyl-1,2,4-oxadiazol-5-yl)aniline (**1**)

a. 1.0 g (7.2 mmol) of 4-aminobenzoic acid was dissolved under an inert atmosphere in 20 mL of DMF, and 1.28 g (7.9 mmol) of CDI solved in 30 mL of DMF was added. After 30 minutes of stirring at room temperature 0.91 g (7.9 mmol) of *tert*-butylamidoxime was added. A second portion of CDI (1.28 g, 7.9 mmol) was added, and the reaction mixture was heated under reflux until the reaction was complete. The mixture was cooled to room temperature and poured into 500 mL of water–ice mixture. The formed solid was filtered off, washed with water, dried and flash chromatographed (silica, ethyl acetate/hexane 2:1), yielding after purification 0.92 g (4.2 mmol, 59%) of **1** as a yellow solid.

b. To a mixture of 0.116 g (1 mmol) of *tert*-butylamidoxime in 5 mL of solvent (acetonitrile or DMF) under an inert atmosphere was added 0.15 g (1 mmol) of 4-nitrobenzonitrile. To this reaction mixture was added 0.3 mmol of acid catalyst 1 (57.0 mg PTSA, 70.8 mg MSA or 28.8 mg MeSA) and 0.3 mmol of catalyst 2 (40.9 mg ZnCl_2_ or 67.5 mg ZnBr_2_), and the mixture was heated to 80 °C until no further change was observed (2–12 hours). Then the reaction mixture was cooled to room temperature, 10 mg of Pt/C 10% catalyst was added, and hydrogen was bubbled through the solution until intermediate **2** was no longer detected. The reaction mixture was filtered through a layer of Celite, and then the solvent was removed under reduced pressure. The solid that remained was dried and flash chromatographed (silica, ethyl acetate/hexane 2:1), yielding after purification **1** as a yellow solid. Yield: PTSA/ZnCl_2_/DMF 139.0 mg, 0.64 mmol, 64%; MSA/ZnCl_2_/DMF 165.0 mg, 0.76 mmol, 76%; MeSA/ZnCl_2_/DMF 121.6 mg, 0.56 mmol, 56%; PTSA/ZnBr_2_/DMF 152.0 mg, 0.7 mmol, 70%; MSA/ZnBr_2_/DMF 171.5 mg, 0.79 mmol, 79%; MeSA/ZnBr_2_/DMF 125.9 mg, 0.58 mmol, 58%; PTSA/ZnCl_2_/CH_3_CN 178.1 mg, 0.82 mmol, 82%; MSA/ZnCl_2_/CH_3_CN 199.8 mg, 0.92 mmol, 92%; MeSA/ZnCl_2_/CH_3_CN 136.8 mg, 0.63 mmol, 63%; PTSA/ZnBr_2_/CH_3_CN 186.7 mg, 0.86 mmol, 86%; MSA/ZnBr_2_/CH_3_CN 201.9 mg, 0.93 mmol, 93%; MeSA/ZnBr_2_/CH_3_CN 141.1 mg, 0.65 mmol, 65%. MS *m*/*z*: 217 (M^+^, 60), 202 (5), 120 (100); HRMS: calcd. for C_12_H_15_N_3_O^+^, 217.12096; found, 217.12159; ^1^H NMR (200 MHz, CDCl_3_) δ 7.87–7.78 (m, 2H), 6.68–6.56 (m, 2H), 4.08 (bs, 2H), 1.32 (s, 9H); ^13^C NMR (50 MHz, CDCl_3_) δ 27.8 (-CH_3_), 31.7, 113.5, 113.7, 129.1, 149.7, 174.7, 177.3; mp 120–122 °C; IR (ATR): 1/λ 3437 (w), 3326 (w), 3218 (w), 2966 (w), 2360 (w), 2162 (w), 1699 (w), 1652 (w), 1603 (s), 1581 (m), 1522 (w), 1510 (m), 1491 (m), 1463 (m), 1442 (m), 1415 (w) 1394 (w), 1351 (s), 1329 (w), 1291 (w), 1197 (m), 1186 (s), 1172 (s), 1096 (w), 1028 (w), 962 (w), 845 (m), 835 (m), 777 (s), 700 (w), 645 (s) cm^−1^.

#### Representative procedure for the synthesis of 3-*tert*-butyl-5-(4-nitrophenyl)-1,2,4-oxadiazole (**2**)

To a mixture of 0.116 g (1 mmol) of *tert*-butylamidoxime in acetonitrile under an inert atmosphere was added 0.150 g (1 mmol) of 4-nitrobenzonitrile in molar ratio 1:1. To this reaction mixture was added 0.3 mmol (0.07 g) of 2-mesitylenesulfonic acid and 0.3 mmol (0.067 g) of ZnBr_2_, and the mixture was heated to 80 °C for 2 hours. After the reaction was finished the heating was removed, and the mixture was cooled to room temperature. The solvent was concentrated under reduced pressure, and ethyl acetate was added. The mixture was washed with sodium hydrogen carbonate solution, water and brine. The organic phase was dried over anhydrous sodium sulfate, filtered, and the solvent was removed. The resulting residue was dried in vacuum and flash chromatographed (silica, ethyl acetate/hexane 2:1), yielding after purification 0.232 g (0.94 mmol, 94%) of **2** as a white solid. MS *m*/*z*: 247 (M^+^, 25), 232 (45), 150 (100); ^1^H NMR (200 MHz, CDCl_3_) δ 8.43–8.28 (m, 4H), 1.45 (s, 9H); ^13^C NMR (50 MHz, CDCl_3_) δ 28.4, 32.6, 124.2, 129.9, 144.6, 148.9, 174.2, 178.4.

#### Synthesis of 4-(4-(3-*tert*-butyl-1,2,4-oxadiazol-5-yl)phenylamino)-4-oxobutanoic acid (**3**)

0.3 g (1.38 mmol) of 4-(3-*tert*-butyl-1,2,4-oxadiazol-5-yl)aniline (**1**) was mixed under inert conditions with an equimolar amount (0.138 g, 1.38 mmol) of succinic anhydride in 20 mL of DCM. This mixture was vigorously stirred at room temperature for 5 hours. The remaining residue was freed from solvent, suspended in 50 mL of water, and the pH value adjusted to 2–3 with a 1 N solution of HCl. The resulting suspension was filtered, and the product was dried in vacuum. It was purified by flash chromatography (silica gel, ethyl acetate/hexane 1:1), yielding 0.398 g (1.25 mmol, 91%) of **3** as a white solid after purification. MS *m*/*z*: 317 (M^+^, 15), 299 (55), 217 (50), 202 (100), 120 (55); HRMS: calcd. for C_16_H_19_N_3_O_4_^+^, 317.13701; found, 317.13717; ^1^H NMR (200 MHz, DMSO-*d*_6_) δ 12.24 (bs, 1H), 10.42 (s, 1H, -NH), 8.07–8.02 (m, 2H), 7.96–7.92 (m, 2H), 2.67–2.59 (m, 4H), 1.38 (s, 9H); ^13^C NMR (50 MHz, DMSO-*d*_6_) δ 28.1 (-CH_3_), 28.5, 31.1, 31.9, 117.6, 118.8, 128.6, 143.3, 170.7, 173.6, 174.3, 177.5; mp 201–203 °C; IR (ATR): 1/λ 3385 (w), 2964 (w), 1696 (s), 1608 (m), 1594 (w), 1506 (s), 1492 (m), 1464 (w), 1410 (m), 1394 (w), 1351 (m), 1337 (m), 1313 (m), 1281 (w), 1249 (w), 1200 (m), 1177 (m), 1159 (s), 994 (w), 859 (m), 830 (w), 802 (w), 773 (m), 681 (m), 612 (m) cm^−1^.

#### Synthesis of 1-(4-(3-*tert*-butyl-1,2,4-oxadiazol-5-yl)phenyl)pyrrolidine-2,5-dione (**4**)

0.3 g (0.94 mmol) of 4-(4-(3-*tert*-butyl-1,2,4-oxadiazol-5-yl)phenylamino)-4-oxobutanoic acid (**3**) was mixed with an equimolar amount of sodium acetate (0.077 g, 0.94 mmol) in 15 mL of acetic anhydride, and the mixture was then heated for 4 h at 80–85 °C. After cooling, the product was washed with 100 mL H_2_O at pH ≈ 2–3. The solid was dried in vacuum and flash chromatographed (silica, ethyl acetate/hexane 2:1), yielding after purification 0.244 g (0.81 mmol, 87%) of **4** as a white solid. MS *m*/*z*: 299 (M^+^, 55), 284 (15), 202 (100); HRMS: calcd. for C_16_H_17_N_3_O_3_^+^, 299.12644; found, 299.12695; ^1^H NMR (200 MHz, CDCl_3_) δ 8.18–8.09 (m, 2H), 7.48–7.39 (m, 2H), 2.80 (s, 4H), 1.34 (s, 9H); ^13^C NMR (50 MHz, CDCl_3_) δ 28.3 (-CH_3_), 32.3, 124.2, 126.6, 128.6, 135.3, 174.0, 175.5, 178.3; mp 136–138 °C; IR (ATR): 1/λ 2970 (w), 2875 (w), 1776 (w), 1709 (s), 1613 (w), 1520 (w), 1500 (w), 1465 (w), 1419 (w), 1382 (m), 1351 (w), 1275 (w), 1197 (m), 1171 (s), 923 (w), 904 (w), 841 (m), 810 (w), 771 (w), 737 (w), 697 (m), 659 (w) cm^−1^.

#### Synthesis of methyl 4-(4-(3-*tert*-butyl-1,2,4-oxadiazol-5-yl)phenylamino)-4-oxobutanoate (**5**)

A suspension of 0.3 g (0.94 mmol) of 4-(4-(3-*tert*-butyl-1,2,4-oxadiazol-5-yl)phenylamino)-4-oxobutanoic acid (**3**) in 10 mL of diethyl ether was cooled in an ice bath, and a solution of diazomethane in diethyl ether 2.5 N was added dropwise until the white solid disappeared, and the gas evolution stopped. The solvent was removed under high vacuum to afford 0.298 g (0.9 mmol, 96%) of **5** as a white solid. No further purification was necessary. MS *m*/*z*: 331 (M^+^, 10), 299 (50), 284 (15), 202 (100); HRMS: calcd. for C_17_H_21_N_3_O_4_^+^, 331.15266; found, 331.15275; ^1^H NMR (200 MHz, CDCl_3_) δ 8.34 (s, 1H-NH), 8.11–8.02 (m, 2H), 7.70–7.66 (m, 2H), 3.72 (s, 3H), 2.82–2.67 (m, 4H), 1.42 (s, 9H); ^13^C NMR (50 MHz, CDCl_3_) δ 27.8 (-CH_3_), 28.3, 31.3, 31.7, 51.4, 118.7, 119.3, 128.4, 141.0, 169.5, 173.0, 173.9, 177.6; mp 144–146 °C; IR (ATR): 1/λ 3391 (w), 2966 (w), 1738 (m), 1702 (m), 1607 (m), 1595 (w), 1507 (s), 1492 (m), 1524 (w), 1392 (w), 1351 (m), 1327 (s), 1274 (w), 1251 (w), 1197 (m), 1172 (s), 1156 (s), 1097 (w), 991 (w), 988 (w), 863 (m), 836 (w), 797 (w), 773 (m), 699 (w), 690 (w), 617 (s) cm^−1^.

#### Synthesis of (*Z*)-4-(4-(3-*tert*-butyl-1,2,4-oxadiazol-5-yl)phenylamino)-4-oxobut-2-enoic acid (**6**)

0.3 g (1.38 mmol) of 4-(3-*tert*-butyl-1,2,4-oxadiazol-5-yl)aniline (**1**) was mixed under inert conditions with an equimolar amount of 0.135 g (1.38 mmol) of maleic anhydride in 20 mL of DCM. This mixture was vigorously stirred at room temperature for 3 hours. The remaining residue was freed from solvent and suspended in 50 mL of water, and the pH value was adjusted to 2–3 with a 1 N solution of HCl. The resulting suspension was filtered, the product dried in vacuum and flash chromatographed (silica, ethyl acetate/hexane 2:1), yielding after purification 0.365 g (1.15 mmol, 84%) of **6** as a white solid. MS *m*/*z*: 315 (M^+^, 15), 297 (50), 217 (55), 200 (100), 120 (75); HRMS: calcd. for C_16_H_17_N_3_O_4_^+^, 315.12136; found, 315.12144; ^1^H NMR (200 MHz, DMSO-*d*_6_) δ 12.94 (bs, 1H), 10.73 (s, 1H, -NH), 8.14–8.03 (m, 2H), 7.96–7.85 (m, 2H), 6.61–6.32 (m, 2H), 1.38 (s, 9H); ^13^C NMR (50 MHz, DMSO-*d*_6_) δ 28.0 (-CH_3_), 31.9, 118.3, 119.4, 128.7, 130.1, 131.4, 142.7, 163.6, 166.8, 174.2, 177.5; mp 214–216 °C; IR (ATR): 1/λ 3288 (w), 3104 (w), 2974 (w), 1707 (m), 1633 (w), 1616 (w), 1586 (m), 1566 (w), 1532 (s), 1520 (s), 1496 (s), 1467 (m), 1409 (w), 1396 (w), 1348 (w), 1325 (m), 1281 (w), 1267 (w), 1188 (m), 1101 (w), 976 (m), 899 (w), 849 (s), 776 (m), 737 (w), 738 (w), 632 (w), 615 (m) cm^−1^.

#### Synthesis of 1-(4-(3-*tert*-butyl-1,2,4-oxadiazol-5-yl)phenyl)-1*H*-pyrrole-2,5-dione (**7**)

0.25 g (0.79 mmol) of (*Z*)-4-(4-(3-*tert*-butyl-1,2,4-oxadiazol-5-yl)phenylamino)-4-oxobut-2-enoic acid (**6**) was mixed with an equimolar amount of sodium acetate (0.065 g, 0.79 mmol) in 15 mL of acetic anhydride, and the mixture was then heated for 4 h at 80–85 °C. After cooling, the product was washed with 100 mL of water at pH ≈ 2–3. The solid was dried in vacuum and flash chromatographed (silica, ethyl acetate/hexane 2:1), yielding after purification 0.176 g (0.59 mmol, 75%) of **7** as a white solid. MS *m*/*z*: 297 (M^+^, 60), 282 (15), 200 (100); HRMS: calcd. for C_16_H_15_N_3_O_3_^+^, 297.11079; found, 297.11127; ^1^H NMR (200 MHz, CDCl_3_) δ 8.26–8.14 (m, 2H), 7.62–7.54 (m, 2H), 6.88 (s, 2H), 1.44 (s, 9H); ^13^C NMR (50 MHz, CDCl_3_) δ 28.2 (-CH_3_), 32.3, 123.2, 125.5, 128.5, 134.2, 134.8, 168.6, 174.0, 178.3; mp 123–125 °C; IR (ATR): 1/λ 3474 (w), 3096 (w), 2975 (w), 2927 (w), 1713 (s), 1614 (w), 1567 (w), 1521 (m), 1502 (m), 1466 (w), 1395 (m), 1385 (m), 1351 (w), 1306 (w), 1238 (w), 1195 (m), 1149 (s), 1064 (w), 1022 (w), 988 (w), 949 (w), 905 (w), 842 (m), 827 (s), 771 (m), 737 (w), 700 (s), 686 (m), 652 (w), 624 (w) cm^−1^.

### X-ray structure determinations

Data are summarized in Table 2. Intensities were determined at 100 K on Oxford Diffraction diffractometers by using monochromated Mo Kα or mirror-focussed Cu Kα radiation. Structures were refined on *F*^2^ using the program SHELXL-97 [38]. Hydrogen atoms were refined either freely (NH), by using rigid methyl groups allowed to rotate but not tip or by using a riding model starting from calculated positions. Structure **5** was treated as a non-merohedral twin.

CCDC–924911 (**1**), CCDC–951265 (**2**), CCDC–924912 (**4**), CCDC–924913 (**5**), CCDC–924914 (**6**) contain the supplementary crystallographic data for this paper. These data can be obtained free of charge from the Cambridge Crystallographic Data Centre via http://www.ccdc.cam.ac.uk/.

**Table 2:** Crystallographic data for compounds **1**, **2**, **4**–**6**.

Compound	**1**	**2**	**4**	**5**	**6**

					
Formula	C_12_H_15_N_3_O	C_12_H_137_N_3_O_3_	C_16_H_17_N_3_O_3_	C_17_H_21_N_3_O_4_	C_17_H_21_N_3_O_4_
*M*_r_	217.27	247.25299.33	299.33	331.37	331.37
Habit	colourless tablet	colourless tablet	colourless needle	colourless plate	colourless prism
Cryst. size (mm)	0.15 × 0.15 × 0.1	0.24 × 0.12 × 0.07	0.11 × 0.05 × 0.02	0.18 × 0.09 × 0.02	0.07 × 0.05 × 0.03
Crystal system	monoclinic	orthorhombic	monoclinic	monoclinic	trigonal
Space group	P2_1_/c	Pbca	P2_1_/c	*P* 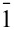	*R* 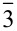
Cell constants:					
*a* (Å)	15.6514(10)	6.7175(4)	13.8569(4)	5.4494(5)	28.0192(6)
*b* (Å)	9.0961(6)	13.8116(6)	15.3562(4)	6.1403(6)	28.0192(6)
*c* (Å)	18.4999(12)	26.1018(9)	6.8926(2)	25.283(2)	10.0740(8)
α (°)	90	90	90	86.906(8)	90
β (°)	114.371(8)	90	94.918(2)	85.277(8)	90
γ (°)	90	90	90	72.926(8)	120
*V* (Å^3^)	2399.1	2421.7	1461.27	805.57	6849.3
Z	8	8	4	2	18
*D*_x_ (Mg m^−3^)	1.203	1.356	1.361	1.366	1.376
μ (mm^−1^)	0.08	0.10	0.80	0.81	0.84
*F*(000)	928	1040	632	352	2988
*T* (°C)	−173	−173	−173	−173	−173
Wavelength (Å)	0.71073	0.71073	1.54184	1.54184	1.54184
2θ_max_	52.8	56.6	152	152	152
Refl. measured	41755	55696	16680		28530
Refl. indep.	4908	2483	3034	3747	3156
*R*_int_	0.064	0.074	0.027		0.067
Parameters	311	166	202	226	219
*wR*(*F*^2^, all refl.)	0.057	0.061	0.086	0.179	0.098
*R*(*F*, >4σ(*F*))	0.035	0.031	0.032	0.062	0.039
S	0.73	0.81	1.06	1.07	1.04
max. Δ/ρ (e Å^−3^)	0.17	0.18	0.21	0.38	0.17

### In vitro antitumor activity toward human tumor cell lines

Antitumor activity of the compounds was tested in a monolayer cell survival and proliferation assay with human tumor cell lines. Studies carried out with panels of human tumor cell lines of different origin and histotype allow for the analysis of potency and tumor selectivity of test compounds.

Ten out of the eleven tested cell lines were established at Oncotest from patient-derived human tumor xenografts passaged subcutaneously in nude mice [39]. The origin of the donor xenografts was described [40–41]. The cell line HT-29 was kindly provided by the National Cancer Institute (Bethesda, MA, USA). Cells were cultured in RPMI 1640 medium, supplemented with 10% fetal calf serum and 0.1 mg/mL gentamicin under standard conditions (37 °C, 5% CO_2_). Authenticity of all cell lines was proven by STR analysis at the DSMZ (Braunschweig, Germany).

A modified propidium iodide assay was used to assess the compounds’ activity toward human tumor cell lines [42]. Briefly, cells were harvested from exponential phase cultures by trypsinization, counted and plated in 96-well flat-bottom microtiter plates at a cell density dependent on the cell line (4.000–20.000 cells/well). After 24 h recovery period to allow the cells to adhere and resume exponential growth, compounds were added at 10 concentrations in half-log increments and incubated for 4 days. The inhibition of proliferation was determined by measuring the DNA content with an aqueous propidium iodide solution (7 μg/mL). Fluorescence was measured by using a Cytofluor micro-plate reader (excitation λ = 530 nm, emission λ = 620 nm) and thus establishing a direct relationship to the total viable cell number. In each experiment, all measurements were carried out twice.
